# Predictors of hospital stay and mortality in dengue virus infection-experience from Aga Khan University Hospital Pakistan

**DOI:** 10.1186/1756-0500-7-473

**Published:** 2014-07-27

**Authors:** Muhammad Abdul Mabood Khalil, Jackson Tan, Muhammd Ashhad Ullah Khalil, Safia Awan, Manickam Rangasami

**Affiliations:** 1 Department of Medicine, Aga Khan University Hospital, Karachi 74800, Pakistan; 2 Department of Nephrology RIPAS Hospital, Bander Seri Begawan BA1710, Brunei Darussalam; 3 Department of Medicine Khyber Teaching Hospital, Peshawar 25000, Pakistan; 4 Department of Nephrology SSB Hospital, Kuala Belait KA 1131, Brunei Darussalam

**Keywords:** Dengue, Predictors of hospital stay, Mortality

## Abstract

**Background:**

Dengue virus infection (DVI) is very common infection. There is scarcity of data on factor associated with increased hospital stay and mortality in dengue virus infection (DVI). This study was done to know about factors associated with increased hospital stay and mortality in patients admitted with DVI.

**Results:**

Out of 532 patients, two third (72.6%) had stay ≤3 days while one third (27.4%) had stay greater than 3 days. The mean length of hospital stay was 3.46 ± 3.45 days. Factors associated with increased hospital stay (>3 days) included AKI (acute kidney injury) (Odd ratio 2.98; 95% CI 1.66-5.34), prolonged prothrombin time (Odd ratio 2.03; 95% CI 1.07-3.84), prolonged activated partial thromboplastin time (aPTT) (Odd ratio 1.80; CI 95% 1.15-2.83) and increased age of > 41.10 years (Odd ratio 1.03; CI 95% 1.01-1.04).Mortality was 1.5%. High mortality was found in those with AKI (P <0.01), dengue hemorrhagic fever (DHF) and dengue shock syndrome (DSS) (P <0.001), respiratory failure (P0.01), prolong PT (P 0.001), prolong aPTT (P0.01) and increased hospital stay (P0.04).

**Conclusion:**

Increasing age, coagulopathy and acute kidney injury in patients with DVI is associated with increased hospital stay. Morality was more in patients with AKI, DHF and DSS, respiratory failure, coagulopathy and these patients had more prolonged hospitalization.

## Background

Dengue virus infection (DVI) is mosquito born disease and is a growing health problem in the world. Due to the advent increase in trade activities and tourism, the virus has been inadvertently transferred from endemic regions to other parts of the world [1,2]. The number of countries reporting DVI has increased from nine in 1950 to over 100 countries over the past few decades. As a result, over 50 million people are infected by DVI and around 2.5 billion people are at risk [3].

Dengue virus infection causes significant morbidity and mortality. There are four serotypes (DEN 1–4), classified according to biological and immunological criteria. The virus is transmitted by mosquitoes. The clinical manifestations vary in presentation from mild constitutional symptoms to severe infections causing dengue hemorrhagic fever (DHF) and dengue shock syndrome (DSS). Asymptomatic infections are mild and can be treated as an outpatient. DSS and DHF, on the other hand require admission and treatment in special units equipped with intensive facilities. Dengue was first documented in 1982 in Pakistan [4]. Pakistan had multiple epidemics since the first literature documented epidemic in 1994 [5]. The virus is now endemic in all provinces of the country [6,7]. Recent epidemics in Pakistan had caused significant burden and stress on health care facilities. Dengue is associated with significant morbidity and mortality. Both DSS and DHF have bad expected outcome if not vigilantly picked up and treated. The identification of factors associated with prolong hospital stay and mortality can potentially identify sick patients who can be treated with supportive measures in a special care set up. This in turn can potentially reduce morbidity and mortality. Identification of these risk factors can be incorporated into patient assessment and management protocol to provide better care. To date, there is no literature evidence on length of hospital stay and factors associated with increased hospital stay and mortality. With this in mind, this study was designed to investigate and identify potential factors that may determine length of hospital stay and mortality in patients with DVI.

## Methods

### Design

This retrospective study was conducted at Aga Khan University Hospital Karachi Pakistan.

#### Study setting

Aga Khan University Hospital is a tertiary care health facility located in the biggest city in Pakistan. It has 15 inpatient units with a total capacity of 563 beds and is also the tertiary referral center for advance diagnostic procedures and management. Study was conducted at the department of medicine taking care of adult patients.

### Study population and sampling

Study subjects included in-patients admitted with a primary diagnosis of DVI and were identified from computerized records from January 2008 to December 2010. Study subjects included patients with age greater than 14 years admitted with the primary diagnosis of DVI and confirmed dengue IgM antibodies confirmed by ELISA, irrespective of severity of DVI. Patients with no laboratory evidence of DVI and those with malaria were excluded. Data on demographics, clinical features, laboratory data, length of stay and mortality were noted on a proforma.

### Study tools and data management

Records of the cases were retrieved through codes using international classification of disease (ICD) 9 CM 061 for DVI and international classification of disease (ICD) 9 CM 065.4 for DHF and DSS. Most studies have reported median hospital stay of 3-4 days in patients with DVI [8,9]. Therefore, we divided the length of hospital stay divided into less than or equal to 3 days and greater than 3 days and these two cohorts were compared for variables that might determine predictors for increased hospital stay. Similar comparisons were done between patients who survived and those who died. WHO 1997 classification was used to classify dengue virus infection into dengue fever (DF), dengue hemorrhagic fever (DHF) and dengue shock syndrome (DSS) [10]. We used Acute Kidney Injury Network (AKIN) definition for classification of AKI [11]. AKIN stage 1 AKI was defined as an increase in serum creatinine greater than 26.5 μmole/litre (≥0.3 mg/dl) or 1.5 to 2 fold increase from baseline. AKIN stage 2 AKI was defined as an increase in serum creatinine of greater than 2-3 folds from baseline. AKIN stage 3 AKI was defined as an increase in serum creatinine of greater than 3 fold from baseline or an absolute serum creatinine of greater than 354 μmole/litre (≥4 mg/dl). Mild hepatitis was defined as ALT 45-300 IU/L and severe hepatitis was defined as ALT >300 IU/L.

### Ethical consideration

Ethical approval was taken from both departmental and hospital Ethical Review Committee.

### Statistical analysis

Descriptive statistics were used to summarize baseline values and demographic data. Quantitative and qualitative data were expressed as mean, median, interquartile range (IQR) and standard deviation (SD) and number of observations with percentage (%) respectively. To evaluate the association between outcomes (length of hospital stay) and each of factors, *x*^2^ -test or Fisher’s exact tests of independence were used to compare proportions where appropriate, and the Student’s t-test was used to analyze continuous data. Odds Ratios (OR) and their 95% Confidence Intervals (CI) were estimated using binary logistic regression, with length of hospital stay (≤3 versus >3 days) as outcomes. Multivariable models were constructed, including variables that showed an effect in the prediction of length of hospital stay >3 days in the univariate analysis. All p-values were based on two-sided tests and significance was set at a p-value less than 0.05. The analyses were performed using SPSS (Statistical Package of Social Sciences) version 19.

## Results

Of the 532 patients, two thirds (70.9%) were male. The mean age was 35.2 ± 14.7 years (range 15-85 years). Dengue fever was found in majority of the patients (84.4%). DHF and DSS were present only in 76 (14.3%) and 7 (1.3%) patients respectively. All cases of dengue fever, DHF and DSS were dengue IgM antibodies positive by ELIZA. Seventy one (13.3%) patients developed AKI. Mild and severe hepatitis were present in 329 (61.8%) and 47 (8.8%) patients respectively. Prolongation of prothombin time was found in 64 (12%) while 225 (42.3%) patients had prolonged aPTT. Fourteen (2.6%) patients had respiratory failure and 11 (2.1%) patients had neurological involvement (Table 1).

**Table 1:** Characteristics of the dengue virus infection cases (n = 532)

**Variables**	**Mean ± standard deviation**	**Median/Range/Percentages**
Age, in years	35.29 ± 14.70	32(15-85)
Gender		
Female	155	29.1%
Male	377	70.9%
Length of hospital stay	3.46 ± 3.45	3(1-33 days); 3[2-4]
Peak creatinine (μmole/litre)	98.124 ± 83.98	79.56(17.68-875.16)
Peak creatinine in AKI (μmole/litre)	229.84 ± 174.15	149.6(114.92-875.16)
Admission creatinine (μmole/litre)	90.16 ± 61.88	79.56(17.68-857.48)
Admission hematocrit (Proportion of 1.0)	0.4102 ± 0.063	0.415(0.199-0.58)
Peak hematocrit (Proportion of 1.0)	0.422 ± 0.0563	0.424(0.264-0.58)
Platelets	38.65 ± 42.14	23(2-427)
Alanine aminotransferase (μkat/L)	2.73 ± 5.86	1.27(0.050-60.55)
Prothrombin time (Seconds)	12.82 ± 8.70	11.10(3-120)
aPTT (Seconds)	36.02 ± 15.12	33.35(11.2-120)
Vasopressin	5	0.9%
Central nervous system involvement	11	2.1%
Respiratory failure	14	2.6%
World Health Organization Classification		
Dengue fever	449	84.4%
Dengue hemorrhagic fever	76	14.3%
Dengue shock syndrome	7	1.3%
Length of stay in hospital		
≤3 days	386	72.6%
>3 days	146	27.4%
Alanine aminotransferase		
Normal	156	29.3%
Mild hepatitis 0.75 - 5.01 μkat/L	329	61.8%
Severe hepatitis >5.01 μkat/L	47	8.8%
Prothrombin time (Seconds)		
≤15	468	88%
>15	64	12%
Activated partial thromboplastin time (Seconds)		
≤ 35	307	57.7%
>35	225	42.3%
Platelets (Micro/liter)		
<50000	406	76.3%
50000-100000	93	17.5%
100000-150000	23	4.3%
>150000	10	1.9%
Number of dengue cases/years		
2008	76	14.3%
2009	148	27.8%
2010	308	57.9%

The mean hospital stay was 3.46 days ± 3.45; Median 3 [IQR; 2-4]. Three hundred and eighty six patients (72.6%) had a length of stay ≤3 days. One hundred and forty six patients (27.4%) had a length of stay > 3 days. The total numbers of patients with DVI progressively increased from 2008 to 2010 (Table 1). Potential determining factors associated in both groups were analyzed through binary logistic regression analysis. AKI was found an independent predictor for increased length of stay greater than 3 days (Odd ratio 2.98; 95% CI 1.66-5.34). Other independent predictors for increased length of stay greater than 3 days included prolong prothrombin time (PT) (Odd ratio 2.03; 95% CI 1.07-3.84), prolong activated partial thromboplastin time (aPTT) (Odd ratio 1.80; 95% CI 1.15-2.83) and increasing age (Odd ratio 1.03; 95% CI 1.01-1.04) (Table 2). Although univariate analysis showed respiratory failure (P <0.001), neurological involvement (P = 0.04) and hepatitis (P value 0.02) were significant associated with increased hospital stay, however these finding were not reciprocated by multivariate analysis (Table 3).

**Table 2:** Predictors of mortality

**Variable**	**Univariate analysis**		
	**Outcome**		**P value**
	**Discharge n = 524**	**Death n = 8**	
Age, in years	35.0 ± 14.5	49 ± 21.6	0.11
Acute kidney injury	63(12)	8(100)	<0.001
World Health Organization Classification			
Dengue fever	447(85.3)	2(25)	<0.001
Dengue hemorrhagic fever	73(13.9)	3(37.5)	
Dengue shock syndrome	4(0.8)	3(37.5)	
Central nervous system	10(1.9)	1(12.5)	0.15
Diabetes mellitus	45(8.6)	2(25)	0.15
Hypertension	46(8.8)	2(25)	0.15
Respiratory failure	12(2.3)	2(25)	0.01
Prothrombin time (Seconds)			
<15	465(88.7)	3(37.5)	0.001
>15	59(11.3)	5(62.5)	
Activated partial thromboplastin time (Seconds)			
<35	306(58.4)	1(12.5)	0.01
>35	218(41.6)	7(87.5)	
Platelets (Micro/litre)			
<150	514(98.1)	8(100)	0.99
>150	10(1.9)	0	
Length of stay	3.3 ± 3.2	10.6 ± 8.4	0.04
Median [IQR]	3[2-4]	10[2-17.75]	

**Table 3:** Predictors of length of hospital stay in individuals aged ≥ 14 years hospitalized for dengue virus infection (n = 532)

**Variable**	**Univariate analysis**			**Multivariate analysis**	
	**Length of stay in hospital**		**P value**	**Odd ratio [95% CI]**	**P value**
	**≤ 3 days n = 386**	**>3 days n = 146**			
Age, in years	33.10 ± 13	41.10 ± 17.20	<0.001	1.03[1.01-1.04]	<0.001
Acute kidney injury	29(7.5)	42(28.8)	<0.001	2.98[1.66-5.34]	<0.001
World Health Organization Classification					
Dengue fever	329(85.2)	120(82.2)	0.002	1.0	
Dengue hemorrhagic fever	56(14.5)	20(13.7)		0.91[0.49-1.69]	0.76
Dengue shock syndrome	1(0.3)	6(4.1)		2.37[0.23-24.2]	0.46
Central nervous system	5(1.3)	6(4.1)	0.04	0.79[0.17-3.6]	0.76
Diabetes mellitus	28(7.3)	19(13)	0.03	0.84[0.38-1.87]	0.68
Hypertension	26(6.7)	22(15.1)	0.003	1.13[0.50-2.51]	0.76
Respiratory failure	4(1)	10(6.8)	<0.001	3.2[0.76-13.3]	0.11
Alanine aminotransferase					
Normal	110(28.5)	46(31.5)	0.02		
Mild hepatitis	249(64.5)	80(54.8)			
Severe hepatitis	27(7)	20(13.7)			
Prothrombin time (Seconds)					
≤ 15	353(91.5)	115(78.8)	<0.001		
> 15	33(8.5)	31(21.2)		2.03[1.07-3.84]	0.02
Activated partial thromboplastin time (Seconds)					
≤ 35	243(63)	64(43.8)	<0.001		
>35	143(37)	82(56.2)		1.80[1.15-2.83]	0.01
Platelets (Micro/litre)					
<50000	295(76.4)	111(76)	0.12	1.0	
50000-100000	70(18.1)	23(15.8)		0.23[0.04-1.13]	0.07
100000-150000	17(4.4)	6(4.1)		0.31[0.03-1.1]	0.11
>150000	4(1)	6(4.1)		0.36[0.06-1.88]	0.22
Death	2(0.5)	6(4.1)	0.002	0.86[0.13-5.4]	0.87

Mean hospital stay was 10.63 ± 8.434 days in those who died. High mortality (n = 8; 1.5%) was found in those with AKI (P <0.01), dengue hemorrhagic fever (DHF) and dengue shock syndrome (DSS) (P <0.001), respiratory failure (P0.01), prolong PT (P 0.001), prolong aPTT (P0.01) and increased hospital stay (P0.04) (Table 2).

## Discussion

Dengue and DHF have emerged as some of the most important mosquito-borne viral diseases. After multiple epidemics of DVI, dengue is now endemic in all provinces of Pakistan and it is in fact a growing public health problem in Pakistan. Our patient demographics are not dissimilar to other DVI patients reported in the literature. Like other South Asian studies, there is a male preponderance for DVI. Studies from India and Singapore reported male to female ratio of 1.9: 1and 1:0.57 respectively [7,12]. This may be because men are more likely to have better access to healthcare in our society compared to women who are less likely to be taken for care at a hospital when ill. Secondly males may be more exposed to mosquito bites from outdoor sources due to their increased outside home activities.

Regarding length of hospital stay, “one third” of patients with DVI had a prolonged hospital stay. The mean hospital stay reported in our study was 3.46 days. Various studies done at national and international level reported a mean stay of 3.4-6.2 days which are comparable to our results [7-9,12]. We specifically concentrated on this latter group of patients who had a prolonged stay at hospital of greater than 3 days. Presence of AKI, increasing age and coagulopathy were associated with increased hospital stay.

AKI was present in 13.3% patients admitted with DVI. AKI in DF results from hypoperfusion and hypoxia from shock [13]. Various studies showed incidence of AKI in 0.9% to 10.8% [13-15] in patients with DVI and associated with increased mortality [15]. We found the presence of AKI associated with prolonged stay in hospital in patients with dengue virus infection.

Significant number of our study patients showed coagulopathy manifested by abnormality of both extrinsic and intrinsic pathway in patients with DVI. Coagulopathy is now a well-established fact [16,17]. Coagulopathy is multifactorial and may be due to low platelets, deranged PT, aPTT and hepatitis. In addition to AKI we found coagulopathy and increasing age as important predictors associated with increased hospital stay. Although we found hepatitis and thrombocytopenia in a significant number of patients but neither of these had an impact on hospital stay. The mortality rate from our study was 1.5%. Various studies from Pakistan have reported mortality of 2.6%-2.7% in general population infected with DVI [18,19]. International data reports a variable mortality ranging from 0-3.7% [20-23]. However it was interesting to note that all those patients who died had AKI. Similarly mortality was more in those who had a prolong hospital stay, respiratory failure, DHF and DSS, and coagulopathy. However the number of patients died in our cohort was very small (n = 8) to compare with those who survived. Therefore, larger cohorts are needed to study various factors associated with mortality.

Increasing age in combination with AKI and coagulopathy might denote seriously sick patients who can potentially have more morbidity in the form of increased hospital stay. These patients also had more propensity to die. AKI might be due to vascular leakage and coagulopathy in patients with DVI. Therefore the combination of AKI, coagulopathy with increasing age in patients with DVI might put them at risk to develop ischemia of various organs resulting in increased hospital stay. Therefore, these patients should be identified at the earliest and treated preferably in a special care set up to avoid morbidity and mortality. To the best of our knowledge, these factors have never been formally implicated in the literature in increasing hospital stay. The presence of these factors in severely ill patients may have necessitated the use of advanced therapies like renal replacement therapy or ventilator support which would have delayed hospital discharge. These patients were also more likely to have co-existing haemodynamic disturbances and bacterial septicaemia which would have required treatment with inotropes and antibiotics. Furthermore, older patients would have other co-morbidities and have rehabilitational issues which may complicate admissions and extend hospital stay.

We acknowledge that there are several limitations in this study. The retrospective nature of data collection may have biased the findings towards patients who are more unwell and hence more likely to be captured and recorded by the system. We would have liked to grade DHF if the study is done prospectively as it would have enabled us to diagnose early DHF which may have been undetected and unrecorded by our coders. We also acknowledge that criteria for admission and discharges may have been variable among various clinicians taking care of these patients and could interfere with the results of our findings. To asses factors associated with mortality more larger cohort of patients is needed. Moreover, the study population included adults patients and hence its results cannot be generalized to pediatric patients. The study is also limited to one center and may not be representative of the population as a whole.

## Conclusion

Acute kidney injury, coagulopathy and increase age are associated with prolonged hospital stay. Mortality is commoner in those with AKI and prolonged hospital stay. With these in mind we advise extra vigilance and cautionary strategies when encountering DVI patients with these risk factors. More studies are needed to investigate preventive strategies on minimizing the occurrences of these potential deleterious factors.

## Abbreviations

DVI: Dengue virus infection; DHF: Dengue hemorrhagic fever; DSS: Dengue shock syndrome; PT: Prothrombin time; aPTT: Activated partial thromboplastin time; AKI: Acute kidney injury.

## Competing interests

Authors declare that they have no competing interests.

## Authors’ contributions

MAM has made protocol and overall plan of study. MAM has also written the final script. MA has collected data and wrote the initial script under supervision of MAM. JT has contributed by reviewing final script and guided from time to time. SA has written research methodology and helped in data analysis. MR helped a lot by reviewing the whole work and drafting the final manuscript. All authors read and approved the final manuscript.
